# Analysis of Lipid Experiments (ALEX): A Software Framework for Analysis of High-Resolution Shotgun Lipidomics Data

**DOI:** 10.1371/journal.pone.0079736

**Published:** 2013-11-07

**Authors:** Peter Husen, Kirill Tarasov, Maciej Katafiasz, Elena Sokol, Johannes Vogt, Jan Baumgart, Robert Nitsch, Kim Ekroos, Christer S. Ejsing

**Affiliations:** 1 Department of Biochemistry and Molecular Biology, University of Southern Denmark, Odense, Denmark; 2 Zora Biosciences Oy, Espoo, Finland; 3 Institute for Microscopic Anatomy and Neurobiology, Johannes Gutenberg University Mainz, Mainz, Germany; UGent / VIB, Belgium

## Abstract

Global lipidomics analysis across large sample sizes produces high-content datasets that require dedicated software tools supporting lipid identification and quantification, efficient data management and lipidome visualization. Here we present a novel software-based platform for streamlined data processing, management and visualization of shotgun lipidomics data acquired using high-resolution Orbitrap mass spectrometry. The platform features the ALEX framework designed for automated identification and export of lipid species intensity directly from proprietary mass spectral data files, and an auxiliary workflow using database exploration tools for integration of sample information, computation of lipid abundance and lipidome visualization. A key feature of the platform is the organization of lipidomics data in ”database table format” which provides the user with an unsurpassed flexibility for rapid lipidome navigation using selected features within the dataset. To demonstrate the efficacy of the platform, we present a comparative neurolipidomics study of cerebellum, hippocampus and somatosensory barrel cortex (S1BF) from wild-type and knockout mice devoid of the putative lipid phosphate phosphatase PRG-1 (plasticity related gene-1). The presented framework is generic, extendable to processing and integration of other lipidomic data structures, can be interfaced with post-processing protocols supporting statistical testing and multivariate analysis, and can serve as an avenue for disseminating lipidomics data within the scientific community. The ALEX software is available at www.msLipidomics.info.

## Introduction

The lipidome of eukaryotic cells comprises hundreds to thousands of molecular lipid species that constitute and functionalize biomembranes, store metabolic energy in lipid droplets and function as signaling molecules that control cell and organism physiology [1–3]. A key tenet of contemporary mass spectrometry-based lipidomics methodology revolves around the identification and quantification of lipid species on a lipidome-wide scale [4–8]. As such, shotgun lipidomics has emerged as a powerful tool for global lipidome analysis that complements mechanistic studies of lipid metabolism, lipid homeostasis and membrane biology [9–13]. The efficacy of shotgun lipidomics stems from its relative technical simplicity where hundreds of lipid species in sample extracts can be quantitatively monitored at high throughput using direct infusion nanoelectrospray ionization combined with high-resolution Fourier transform mass spectrometry (FT MS) or/and tandem mass spectrometry (MS/MS) [2,14]. Notably, lipidomics analysis on a global scale generates large amounts of (spectral) data that requires software routines for automated lipid identification and quantification, and additional data management for subsequent lipidome visualization and bioinformatics analysis.

Extensive lipidome characterization by shotgun lipidomics can be achieved by executing a systematic program of mass spectrometric analyses of sample extracts in positive and negative ion mode, and by incorporating chemical derivatization procedures to specifically monitor poorly ionizing lipid molecules such as cholesterol [4–7]. Executing such an analytical program generates several mass spectral data files per sample that must be queried for lipid identification, and combined into a single dataset for lipidome quantification and visualization. Numerous software tools have been developed for the identification of lipids: LipidQA[15], LIMSA[16], FAAT[17], lipID[18], LipidSearch[19], LipidView[20], LipidInspector[21] and LipidXplorer[22]. These tools cover a broad range of applications spanning dedicated lipid identification for only certain instrumentations and specific mass analysis routines (MS and MS/MS) to cross-platform software featuring user-specified commands querying spectral data in the open-source .mzXML format. Identified lipid species are typically annotated by a shorthand nomenclature corresponding to the information detail of the mass spectrometric analysis [23,24]. The detection of lipid species by FT MS analysis or by MS/MS analysis for lipid class-specific fragment ions (e.g. *m/z* 184.0733 for phosphatidylcholine (PC) species) supports only “sum composition” annotation (e.g. PC 34:1). In comparison, annotation by the more detailed “molecular composition” (e.g. PC 16:0-18:1) requires MS/MS analysis and detection of structure-specific fragment ions [25]. To support the cataloging of lipid species, the LIPID MAPS Consortium recently developed the “Comprehensive Classification System for Lipids” which outlines an informatics framework for lipidomics [26,27]. Using a classification system enables the design of lipid databases where each lipid species is listed together with a range of accessory lipid features such as lipid category (e.g. glycerophospholipid, sphingolipid, glycerolipid, sterol lipid), lipid class, structural attributes (e.g. number of double bonds, fatty acid chain length), chemical formula, mono-isotopic mass and isotope information. These accessory lipid features can be incorporated into lipidomic data processing routines using database-orientated exploration tools to support computations and visualization of distinct lipidome hallmarks. Notably, none of the currently available software tools comprise streamlined processing routines that integrate lipid intensity data, the accessory lipid features and a full catalog of sample information. 

Here we present a platform for processing, management and visualization of high-content shotgun lipidomics datasets acquired using high-resolution Orbitrap mass spectrometry. The platform features a novel software framework termed ALEX (Analysis of Lipid Experiments) that supports automated identification and export of lipid species intensity directly from proprietary mass spectral data files and the integration of accessory lipid features and sample information into a single output structured in “database table format”. This design supports swift data processing and lipidome visualization across large sample sizes using an auxiliary workflow powered by the database exploration tools: Orange [28] and Tableau Software. To demonstrate the efficacy of the platform, we present a comparative neurolipidomics analysis of cerebellum, hippocampus and S1BF from wild-type and knockout mice devoid of the PRG-1 gene encoding a putative lipid phosphate phosphatase [29].

## Materials and Methods

### Chemicals and lipid standards

Chemicals, solvents and synthetic lipid standards were purchased from Sigma-Aldrich, Rathburn Chemicals, Avanti Polar Lipids and Larodan Fine Chemicals AB.

### Mouse brain tissue sampling

Animal experiments were conducted in strict accordance with German law (in congruence with 86/609/EEC) for the use of laboratory animals and approved by the local animal welfare committee at the Johannes Gutenberg University Mainz. Male C57Bl/6 wild-type and PRG-1 knockout mice [29] were euthanized by an intraperitoneal injection of ketamine at an overdose. Subsequently, the mice were perfused intracardially with 4°C 155 mM ammonium acetate, and the cerebellum, hippocampus and S1BF were dissected. The tissues were immediately frozen on dry ice and stored at -80°C until further processing. 

### Lipid extraction

Brain tissues were homogenized in 155 mM ammonium acetate and analyzed for total protein concentration using BCA Protein Assay Kit (Thermo Scientific). Aliquots of tissue homogenates corresponding to 10 µg of total protein were subjected to lipid extraction executed at 4°C as previously described [7]. Briefly, the tissue homogenates were spiked with 10 μl of internal mixture (providing a total spike of 54 pmol CE 19:0, 35 pmol TAG 17:1/17:1/17:1, 35 pmol DAG 19:0/19:0, 26 pmol LPA O-16:0, 35 pmol PA 17:0/14:1, 25 pmol LPS 17:1, 13 pmol PS 17:0/20:4, 50 pmol PE O-20:0/O-20:0, 30 pmol LPC O-17:0, 137 pmol PC 18:3/18:3, 35 pmol PI 17:0/20:4, 30 pmol PG 17:0/17:0, 55 pmol Cer 18:1;2/17:0;0, 69 pmol SM 18:1;2/17:0;0, 49 pmol HexCer 18:1;2/12:0;0, 28 pmol SHexCer 18:1;2/12:0;0) and diluted to 200 μl using 155 mM ammonium acetate. Samples were subsequently added 990 µl chloroform/methanol (10:1, v/v) and vigorously mixed for 2 h. The lower organic phase was collected (10:1-phase lipid extract). The remaining aqueous phase was re-extracted with 990 µl of chloroform/methanol (2:1, v/v) for 1 h and the lower organic phase was collected (2:1-phase lipid extract). The collected lower organic phases were vacuum evaporated. 

### Shotgun lipidomics analysis

Lipid extracts were dissolved in 60 μl of chloroform/methanol (1:2, v/v) and subjected to mass spectrometric analysis using an LTQ Orbitrap XL instrument (Thermo Fisher Scientific) equipped with a TriVersa NanoMate (Advion Biosciences) as previously described [4,7]. The 10:1-phase lipid extracts were analyzed by positive ion mode multiplexed FT MS analysis with scan ranges *m/z* 280-580 (monitoring lysophosphatidylcholine (LPC) and lysophosphatidylethanolamine (LPE) species) and *m/z* 500-1200 (monitoring sphingomyelin (SM), ceramide (Cer), diacylglycerol (DAG), PC, ether-linked PC (PC O-), phosphatidylethanolamine (PE), ether-linked phosphatidylethanolamine (PE O-) and triacylglycerol (TAG) species). The 2:1-phase lipid extracts were analyzed by negative ion mode multiplexed FT MS analysis with scan ranges *m/z* 370-660 (monitoring lysophosphatidic acid (LPA), lysophosphatidylserine (LPS) and lysophosphatidylinositol (LPI) species) and *m/z* 550-1700 (monitoring phosphatidic acid (PA), phosphatidylserine (PS), phosphatidylinositol (PI), phosphatidylglycerol (PG) and sulfatide (SHexCer) species). All FT MS spectra were acquired in profile mode using a target mass resolution of 100,000 (fwhm), activation of isolation waveforms, automatic gain control at 1e6, max injection time at 250 ms and acquisition of 2 µscans.

### Annotation of lipid species

Glycerophospholipid and glycerolipid species were annotated using sum composition: <lipid class><total number of C in the fatty acid moieties>:<total number of double bonds in the fatty acid moieties> (e.g. PI 34:1). Sphingolipid species were annotated as <lipid class><total number of C in the long-chain base and fatty acid moiety>:< total number of double bonds in the long-chain base and fatty acid moiety>;< total number of OH groups in the long-chain base and fatty acid moiety> (e.g. SM 36:1;2) [5,7].

### ALEX software

The individual parts of the ALEX software were programmed using several programming languages, libraries and software frameworks. The ALEX lipid database is implemented using the library based SQLite database engine. The ALEX target list generator is written in C++ and uses the Qt framework for its user interface. The ALEX converter, ALEX extractor and ALEX unifier are written in Python 2.7 and make use of the Python packages PySide, NumPy and SciPy. Furthermore, the ALEX converter uses the package comtypes to interface with the MSFileReader library version 2.2 (Thermo Scientific), which must be installed. Finally, the standalone ALEX lipid calculator is written in common lisp and uses the GTK+ framework for its user interface, while the online version is written in PHP. The ALEX software is available at http://mslipidomics.info | software along with installation instructions. A sample dataset is also available for testing local installations of the software.

### Data processing and visualization

Computation of molar abundance (fmol) of lipid species [20] was performed using open source software Orange 2.6 (www.orange.biolab.si) [28]. The Orange workflow is provided as part of the sample dataset available at http://mslipidomics.info | software. In addition, the Orange output with lipidomics data is available as Data S1. Visualization and calculation of mol% values were performed using commercially available Tableau Software (www.tableausoftware.com). Lipidomic data in Tableau file format is available as Data S2 and can be navigated using the freeware Tableau Reader (http://www.tableausoftware.com/products/reader).

## Results and Discussion

### Input: high-resolution shotgun lipidomics data

Shotgun lipidomics platforms based on high-resolution Orbitrap mass spectrometry and automated nanoelectrospray ionization support high throughput analysis with high sensitivity, specificity and extensive lipidome coverage [4,5,7,30]. The extensive lipidome coverage is generated by combined analyses of sample extracts in negative and positive ion mode, and by implementing chemical derivatization procedures to monitor low abundant or poorly ionizing lipid species [31–33]. In order to maximize the sensitivity of Orbitrap mass analysis, we typically record two multiplexed FT MS scans covering a low *m/z* range and high *m/z* range (e.g. +FT MS *m/z* 280-580 and +FT MS *m/z* 500-1200). Each FT MS scan is recorded in profile mode with a target mass resolution of 100,000. The rationale for this multiplexed FT MS analysis is that the instrument injects a user-specified quantum of ions into the Orbitrap for mass analysis (defined by the automated gain control). Hence, multiplexing two or more scan ranges allows separate quanta of ions within specific *m/z* ranges to be analyzed sequentially in the Orbitrap and yields better ion statistics as compared to injecting all ions at the same time when monitoring a wider *m/z* range (e.g. +FT MS *m/z* 200-1200) [34]. We note that the boundaries of the scan ranges should be chosen to cover specific lipid classes and respective internal standards. For example, the scan range +FT MS *m*/*z* 280-580 is used for monitoring LPC and LPE species whereas +FT MS *m/z* 500-1200 analysis is used for monitoring Cer, SM, HexCer, PC, PC O-, PE, PE O-, DAG and TAG species. The total time of analysis is typically set to 3 min. Within this time we record 25 low *m/z* and 25 high *m/z* range FT MS spectra. Likewise, negative ion mode analysis is also executed using multiplexed FT MS acquisitions to monitor negatively charged glycerophospholipid and SHexCer species (see Material and Methods). Consequently, this lipidomics approach generates four distinct mass spectral datasets per sample (two per polarity) that need to be queried for lipid identification and export of lipid species intensity. We note that this lipidomics approach is designed for high throughput-oriented studies and supports annotation of lipid species by sum composition nomenclature (e.g. PC 34:1). Characterization of molecular lipid species (e.g. PC 16:1-18:0) requires implementation of time-consuming MS/MS analysis and lipid identification by dedicated software such as LipidXplorer [22].

### Design of the ALEX software framework

**Figure 1:**
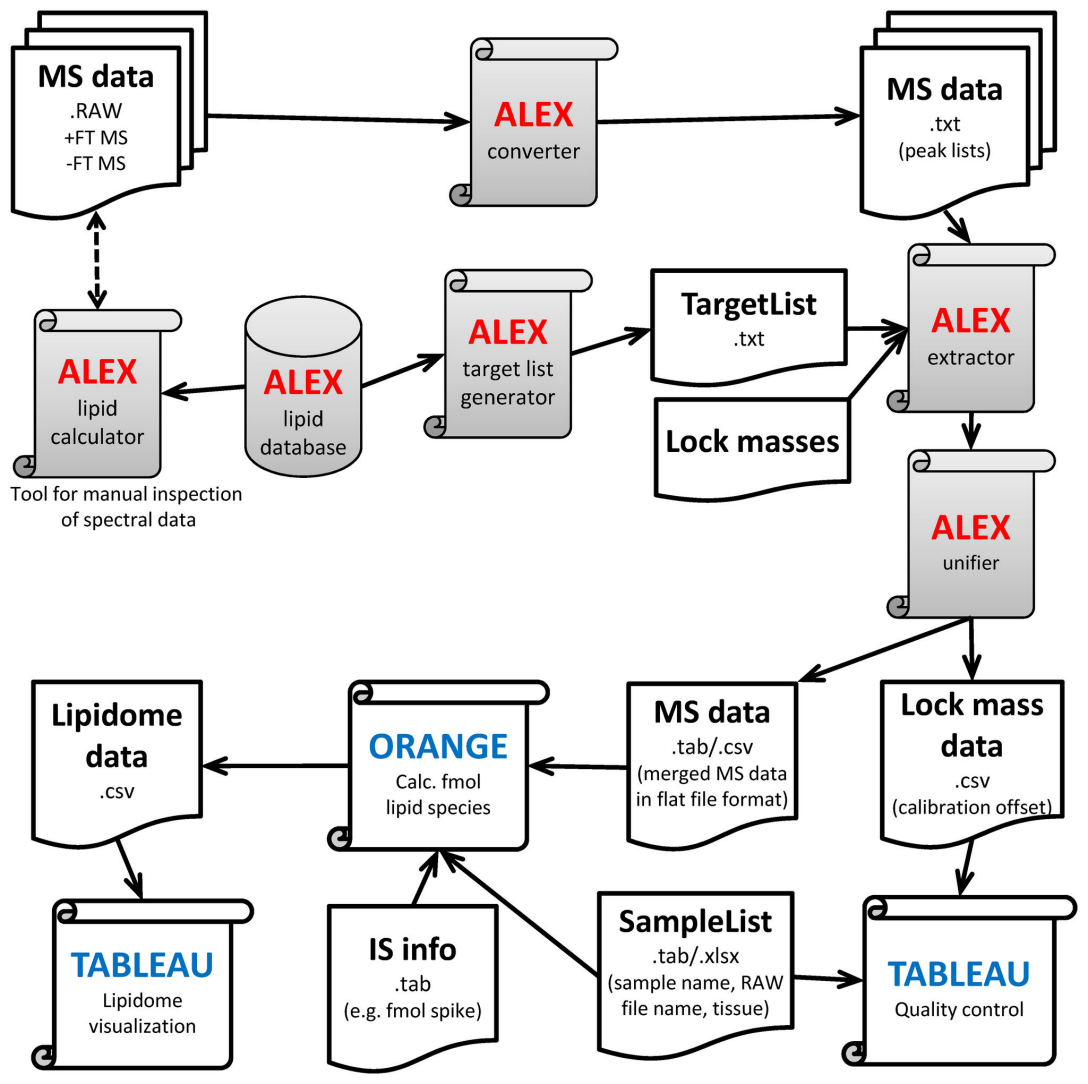
Overview of the ALEX software framework and auxiliary workflow. The ALEX framework comprises six core modules (grey colored boxes). The function of each module is explained in the Results and Discussion section. The output of the ALEX framework includes a data file with identified lipid species, intensities and accessory lipid features across all processed samples and FT MS scan ranges. The ALEX output is organized in database table format that can be accessed and processed by the auxiliary workflow using Orange and Tableau software. The auxiliary workflow is designed to integrate sample information, compute lipid molar abundance, implement quality control procedures and visualize lipidome data.

The ALEX software framework was designed for processing of shotgun lipidomics datasets obtained by multiplexed high-resolution FT MS. The rationales for the design were: i) that ALEX should support lipidomic studies with large sample sets for which a multitude of multiplexed FT MS acquisitions have been acquired; and ii) that the output format of ALEX should be compatible with an auxiliary workflow that supports robust data processing including computation of molar abundances of lipid species across numerous lipid classes, integration of sample information, implementation of data quality control procedures and rapid lipidome visualization. To this end, we designed the ALEX software framework to utilize distinct modules that identify lipid species from proprietary .RAW spectral file format, incorporate accessory lipid features stored in a lipid database and output lipidomic data in “database table format” (Figure 1, Data S1). In this format, the lipidomic data is stored in tabulator or comma separated text files structured as database tables with a separate row for each data point. Each row separately contains fields (also termed attributes) reporting for example the originating sample (.RAW file name), the lipid species, adduct information, intensity, peak area, *m/z* values and accessory lipid features derived from the lipid database. This format also easily supports the management of multiple attributes for each data point (e.g. both peak intensity and area) and can be appended with calculated fields such as lipid abundance (e.g. both pmol and fmol, if needed), and sample information and internal standard information supplied by additional text files in database table format. Importantly, this relational database format provides a robust way to manage large datasets programmatically and avoids the need for error-prone manual alignment of data with lists of accessory features and quantification information. We note that the output of most contemporary lipidomics software tools utilizes a “spreadsheet” format where samples/injections are arranged in the columns and lipid species in the rows of a table of with either intensities or peak areas. This “spreadsheet” format is adequate for processing and visualizing simple lipidomic datasets using tools such as Microsoft Excel, but becomes exceedingly cumbersome if sample sets include more than 10 samples and monitor more than 200 lipid species across different FT MS scan ranges. 

The ALEX software framework consists of six core modules (Figure 1): i) the ALEX lipid database featuring a comprehensive range of lipid species and accessory lipid features used for lipid identification, data processing and management; ii) the ALEX lipid calculator which supports manual interpretation of mass spectra; iii) the ALEX converter which converts mass spectrometric data in proprietary .RAW format into averaged spectral peak lists in text file format; iv) the ALEX target list generator which queries the ALEX lipid database to compile target lists used for lipid species identification; v) the ALEX extractor which uses the target lists to identify lipid species and export corresponding intensities from spectral peak lists; and vi) the ALEX unifier that merges the multiple ALEX extractor outputs containing lipid species data from different FT MS scan ranges into a single data file. Further information about the function of the ALEX modules and the auxiliary workflow is outlined in the subsequent sections.

### ALEX lipid database

To support identification of lipid species we constructed the ALEX lipid database. Currently, the database covers more than 20,000 lipid species from more than 85 lipid classes. Each lipid species in the database is annotated by sum composition and contains a range of accessory lipid features denoting its chemical formula, mono-isotopic mass, adduction in positive and negative ion mode, lipid category, lipid class, and the total number of C atoms, double bonds and hydroxyl group in fatty acid and long chain base moieties (termed C index, db index and OH index, respectively). The ALEX lipid database is available as a collection of text files and as a binary SQLite database queried by the ALEX lipid calculator and the ALEX target list generator, respectively (outlined in the next sections). The text version also serves as the source for the binary version (compiled by a supporting Python program) and can be edited by the user. To support the editing of the database, a template Microsoft Excel file can be used to assist the enumeration and calculation of the chemical formula and mass for each species, such that catalogs of entire lipid classes can readily be added. We note that the accessory lipid features serve as important attributes in the final ALEX output that facilitate data processing and lipidome visualization.

### ALEX lipid calculator

**Figure 2:**
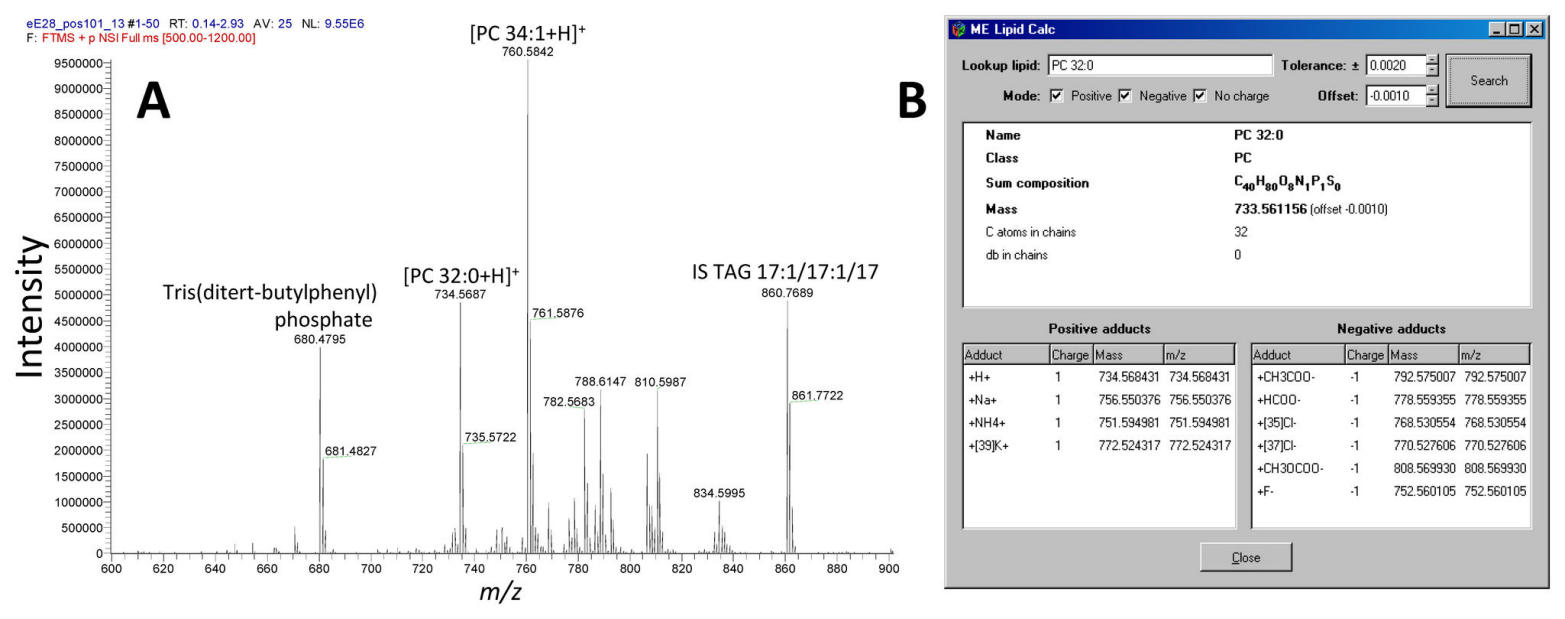
The ALEX lipid calculator. (**A**) Representative positive ion mode FT MS spectrum of a 10:1-phase lipid extract of hippocampus from a PRG-1 knockout mouse. Note that the detection of selected lock mass ions tris(ditert-butylphenyl) phosphate (chemical background, [M+NH_4_]^+^, calculated *m/z* 680.48022, measured *m*/*z* 680.47945, *m*/*z* offset = -0.00077) and TAG 17:1/17:1/17:1 (internal standard (IS), [M+NH_4_]^+^, calculated *m/z* 860.77017, measured *m*/*z* 860.76889 and *m*/*z* offset = -0.00128). The FT MS calibration offset is estimated as the average of the *m/z* offset for both lock mass ions, i.e. the FT MS calibration offset = -0.0010. (**B**) Screenshot of the ALEX lipid calculator showing information for endogenous lipid species PC 32:0 while applying the FT MS calibration offset = -0.0010. Note that the measured *m/z* of PC 32:0 is 734.56872 and that the calculated *m/z* adjusted for the calibration offset is 734.56843 which yield a *m/z* difference of 0.00029 corresponding to a mass error of 0.4 ppm. Without applying lock mass adjustment the mass error would be 1 ppm.

The ALEX lipid calculator was designed to complement manual inspection of FT MS spectra when using proprietary Xcalibur software (Fig. 2). The calculator is available as executable program and as an online application at www.mslipidomics.info/lipid-calc. Both versions of the ALEX lipid calculator support querying *m/z* values of specific lipid species and searching the lipid database for candidate lipid species that match a measured *m/z* value within a user-specified tolerance window. Since the accuracy of lipid identification can be hampered by drifts of the FT MS calibration we also implemented an option to specify an *m/z* offset while querying the lipid database. To accurately specify the calibration offset requires that the user knows the identity of at least one well-characterized “lock mass” ion that can be used for estimating the *m/z* offset (Figure 2). An option to minimize potential problems with FT MS calibration drifts is to apply online lock mass calibration during sample acquisition [35]. However, the online lock mass calibration eliminates the lock mass ion(s) from the recorded FT MS spectra using waveforms that might concomitantly eliminate lipid ions having similar *m/z*. An alternative strategy is to apply an offline lock mass adjustment as implemented in the ALEX extractor (outlined below).

### ALEX converter

A prerequisite for automated lipid identification and export of intensity is that the mass spectrometric data in proprietary .RAW format are made accessible for querying. To this end, we designed the ALEX converter to interface with the proprietary dynamic-link library MSFileReader. The MSFileReader supports export of all scan information within .RAW files including single and averaged spectral peak lists in either centroid or profile mode format. The ALEX converter was designed to export individual spectral peak lists in profile mode format, to average peak lists for specific FT MS scan ranges and to save these averaged peak lists in .txt format. The ALEX converter output consists of a directory with separate folders named according to each FT MS scan range containing corresponding .txt files named according to the originating .RAW files (i.e. the ALEX converter does not merge overlapping FT MS scan ranges). Output files in these FT MS scan range-dependent folders are queried by the ALEX extractor (outlined below). The rationales for this design were i) that no available software supports export of multiplexed FT MS data in profile mode format; and ii) that a simple output text format reporting averaged peak list data allows users to easily review data as in contrast to accessing data in stored in the encrypted .mzXML format. 

### ALEX target list generator

**Figure 3:**
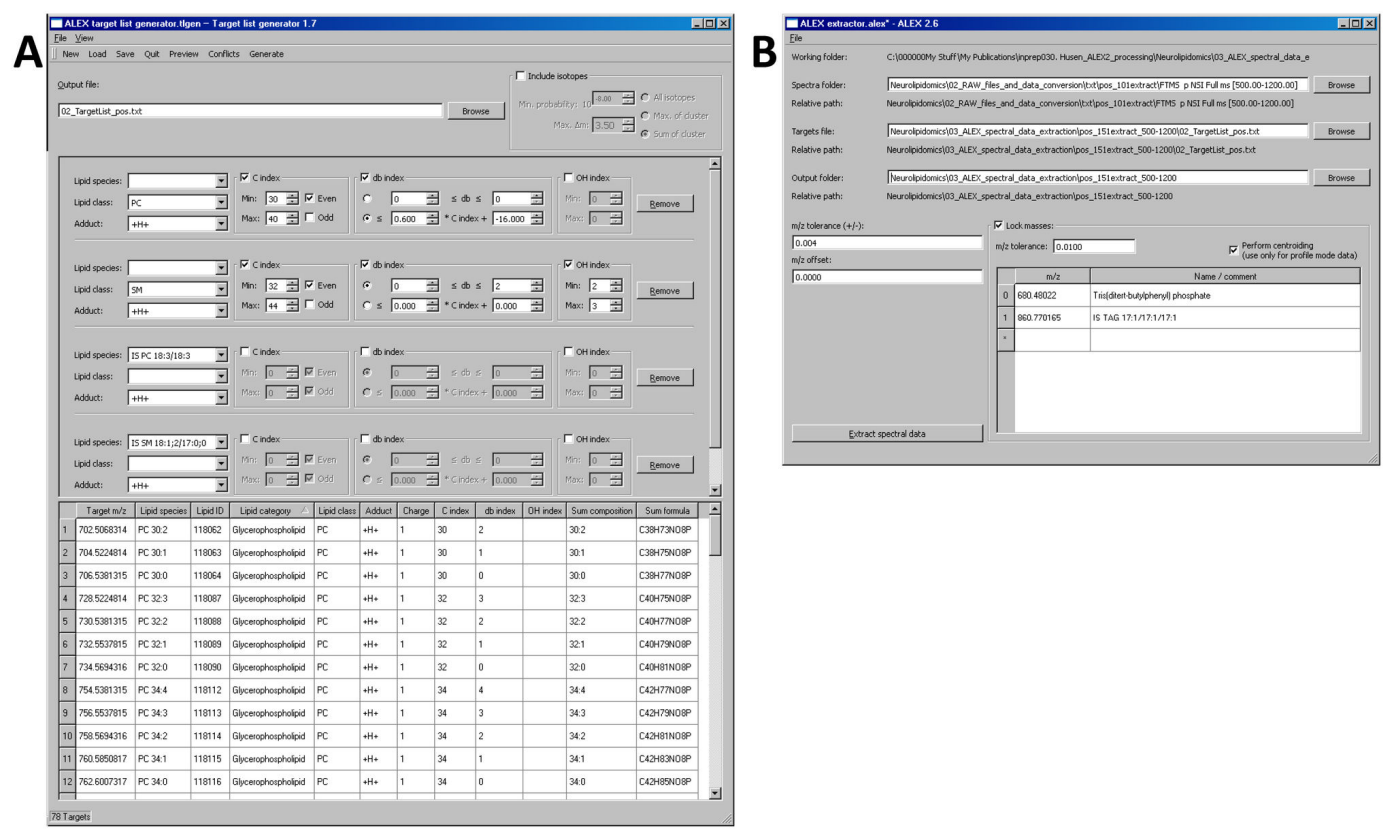
Screenshots of the ALEX target list generator and ALEX extractor. (**A**) The ALEX target list generator allows users to select lipid classes and species to be identified using criteria such as lipid class, adduction, C index, db index and OH index. Individual lipid species including internal standards can also be selected. The ALEX target list generator output is a .txt file with a shortlist of selected lipid species, respective *m/z* values and accessory lipid features. The ALEX target list generator also supports inclusion of isotope information that can be used for deisotoping and isotope correction [20] by applying algorithms within the auxiliary workflow. (**B**) The ALEX extractor identifies lipid species, exports intensity data and incorporates accessory lipid features. As input the ALEX extractor requires the location of spectral peak lists generated by the ALEX converter, a target list compiled by the ALEX target list generator and a location to deposit output files. The ALEX extractor features options to specify an *m/z* tolerance window for lipid identification, to apply a constant *m/z* offset to correct lipid searches for a constant FT MS calibration offset or to apply a lock mass adjustment routine that automatically corrects lipid searches for drifts in FT MS calibration. The automated lock mass adjustment routine requires specification of well-characterized and ubiquitous lock mass ions in order to estimate the FT MS calibration offset.

Lipid identification by the ALEX software framework is based on matching intensity data in the exported peak lists with lipid species information derived from the ALEX lipid database. Important for the design of the identification routine was the ability to accurately identify lipid species, and furthermore to support integration of accessory lipid features and subsequent data management for a multitude of lipid species monitored by various FT MS scan ranges across large sample sets. To this end, the ALEX framework was designed to perform a targeted identification of lipid species and export intensity by querying spectral peak lists using target lists with lipid species and respective *m/z* values. Two modules execute this routine: (i) the ALEX target list generator (Figure 3A) which compiles target lists by querying the ALEX lipid database, and (ii) the ALEX extractor which uses the target lists to identify and extract lipid species intensity (outlined in the next section). We note that a distinct target list with appropriate lipid species should be manually compiled for each FT MS scan range. In addition to listing lipid species and respective *m/z* values, the target lists also include the accessory lipid features derived from the ALEX lipid database. Importantly, these accessory lipid features are incorporated into the final output, and used for processing and visualization by the auxiliary workflow. 

### ALEX extractor and ALEX unifier

**Figure 4:**
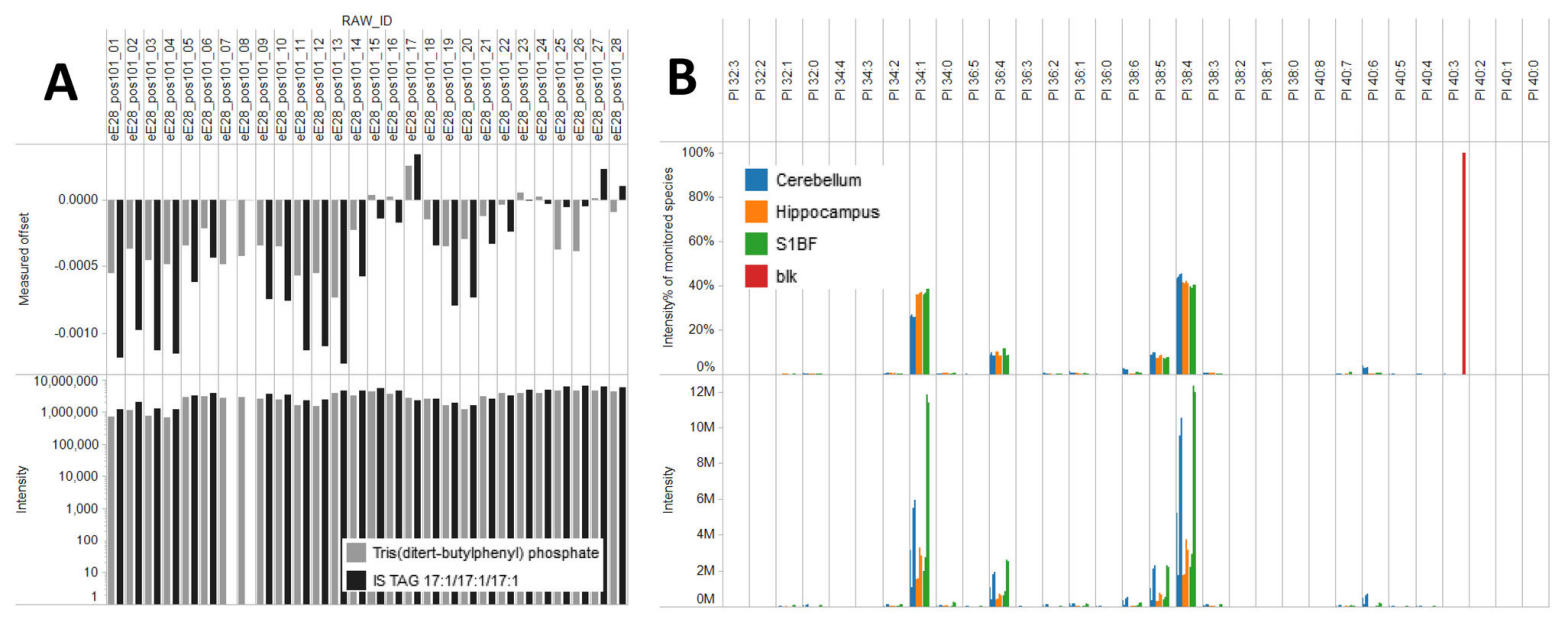
Quality control analysis. (**A**) Monitoring of lock mass offset and lock mass ion intensity as function of sample injection. Notice that the lock mass and internal standard TAG 17:1/17:1/17:1 is not detected in injection 07 and 08. Manual inspection of FT MS spectra revealed that the particular sample had not been spiked with internal standards. (**B**) Assessing the specificity of the PI species profile and intensity across all samples from wild-type mice and the negative control blank samples. Note that in the negative control blank sample (red) a low abundant background ion is detected and falsely identified as PI 40:3. Dubious lipid species can be removed using background subtraction and filtering during subsequent processing in Orange.

The ALEX extractor identifies lipid species and exports intensities by querying the averaged peak lists produced by the ALEX converter (Figure 3B). As input the ALEX extractor requires the folder location containing averaged peak lists, an appropriate target list (i.e. specific for the FT MS scan range) to query the peak lists and a destination folder to deposit output text files. Notably, the ALEX extractor also requires an *m/z* tolerance window to identify lipid species. This *m/z* tolerance window is dependent on instrumental mass resolution and typically set to ±0.0020 amu when processing FT MS data acquired with a target resolution at 100,000. To export lipid species intensity the ALEX extractor selects the maximum intensity value within the specified tolerance window and reports the corresponding *m/z* bin value. As mentioned above, the FT MS calibration can drift during the sample analysis and depending on the time of analysis can yield either a constant calibration offset or a progressively changing offset (see Figure 4A). In order to monitor and adjust lipid searches for potential calibration drifts, we incorporated a novel feature in the ALEX extractor termed “lock mass adjustment”. Lock mass adjustment serves to correct the *m/z* values of targeted lipid species for calibration drifts and thereby support more accurate lipid identification. This lock mass adjustment can be specified as a constant *m/z* offset and applied across all samples being processed. Alternatively, the ALEX extractor features an in-build automatic lock mass adjustment routine that calculates *m/z* calibration offsets for each sample based on selected lock mass ions (Figure 4A). The calculation of calibration offset uses a three-point quadratic interpolation for estimating the centroid *m/z* values of lock mass ions. We note that the automatic lock mass adjustment requires selection of well-characterized and ubiquitous ions as lock masses. For example, by positive ion mode FT MS analysis we always detect both the chemical background ion tris(ditert-butylphenyl) phosphate and the internal standard TAG 17:1/17:1/17:1 (Figure 2). Using these ions as lock masses enables estimation of the FT MS calibration offset for individual samples and correcting lipid searches by adjusting the *m/z* values of targeted lipid species for each sample. As exemplified in Figure 2, using these lock mass ions allows identification of endogenous lipid species with a mass error of 0.4 ppm instead of 1 ppm when ignoring the FT MS calibration offset. Hence, this automated lock mass adjustment routine serves to improve the accuracy of lipid species identification despite drifts in FT MS calibration. 

The ALEX extractor outputs, for each FT MS scan range, several comma-separated value (.csv) files with lipid species intensity data and calculated lock mass adjustments for all processed samples. Notably, the lipid species intensity output is organized in database table format and includes attributes that track the originating .RAW file name, lipid species intensity and peak area, measured *m/z*, calculated *m/z*, the difference between measured and calculated *m/z*, and all the accessory lipid features included on the target list. We note that these sample attributes facilitate subsequent processing and visualization, and implementation of quality control procedures. In order to merge lipid species data from different FT MS ranges, we devised the ALEX unifier to concatenate selected .csv output files into one final .csv file which contains the union of all data and an additional column with an index, “rangeID”, that tracks the FT MS scan range of the input .csv files. This output format supports further data processing including computation of lipid abundance by the auxiliary workflow using open-source Orange [28] and Tableau Software. 

### Application of the ALEX software framework

In order to demonstrate the efficacy of the ALEX software framework and describe the auxiliary workflow, we here present a neurolipidomic pilot study. Three brain tissues; cerebellum, hippocampus and S1BF from two control mice and two mice devoid of the PRG-1 gene were subjected to shotgun lipidomics analysis. Homogenates of the tissues (12 samples in total) and two blank samples were spiked with defined amounts of internal lipid standards and subjected to 2-step lipid extraction [7]. The apolar (10:1-) and polar (2:1-phase) lipid extracts were analyzed by multiplexed FT MS analysis in positive and negative ion mode, respectively. Each sample extract was analyzed twice (technical replicates). In total, this analysis produced 56 .RAW files (14 samples analyzed twice per polarity). 

First, the ALEX converter was used to convert .RAW files to spectral peak lists (Figure 1). This processing produced a total of 112 peak list files (56 .RAW files, two FT MS scan ranges per polarity). The peak list files were automatically organized into 4 folders named according to polarity and scan range: +FTMS *m/z* 280-580, +FTMS *m/z* 500-1200, -FTMS *m/z* 370-660, and -FTMS *m/z* 550-1700 (each folder having 28 peak list files). Next, target lists with lipid species for each of the four FT MS *m/z* ranges were generated using the ALEX target list generator (See Materials and Methods for details). These target lists were subsequently used by the ALEX extractor to identify lipid species and export corresponding intensities from the peak list files. In addition, the processing by the ALEX extractor was performed using lock mass ions for each FT MS *m/z* range in order to monitor and correct searches for FT MS calibration drifts (Figure 4A). Finally, the ALEX unifier was applied to merge the four ALEX extractor output files into single output files reporting all identified lipid species, intensities and accessory lipid features, and lock mass information and calculated FT MS calibration offsets. 

As a first step in the auxiliary workflow we performed a quality control of the neurolipidomics dataset (Figure 1). To this end, we accessed the lock mass information using Tableau Software and displayed the estimated FT MS calibration offsets as function of sample injection. This quality control showed that the calibration offset was not constant across all samples (Figure 4) and thus highlighting the efficacy of the automatic lock mass adjustment. Importantly, for one sample we observed no intensity of the selected lock mass ion TAG 17:1/17:1/17:1. By manual inspection of the .RAW data, we concluded that the investigator in charge had failed to spike internal lipid standards into the particular sample. Consequently, this quality control demonstrated that the particular sample (cerebellum from a knockout mouse) could not be used for computing the molar amount of lipid species. As an additional quality control procedure we also accessed the output file with lipid species intensity data using Tableau Software, and displayed both the absolute intensity and intensity profile of monitored lipid species within lipid classes for all samples analyzed (Figure 4B). This analysis showed that a low abundant ion in the blank samples was falsely identified as PI 40:3. Due to the low intensity of this ion and its presence in the blank samples we concluded that this ion represents a chemical background ion. To minimize bias from falsely identified background ions one can implement a background subtraction during the subsequent computation of molar lipid abundance using the Orange software. Moreover, it is recommendable to perform additional tandem mass analysis to assess the identity of dubious identification.

### Outlining the auxiliary workflow

**Figure 5:**
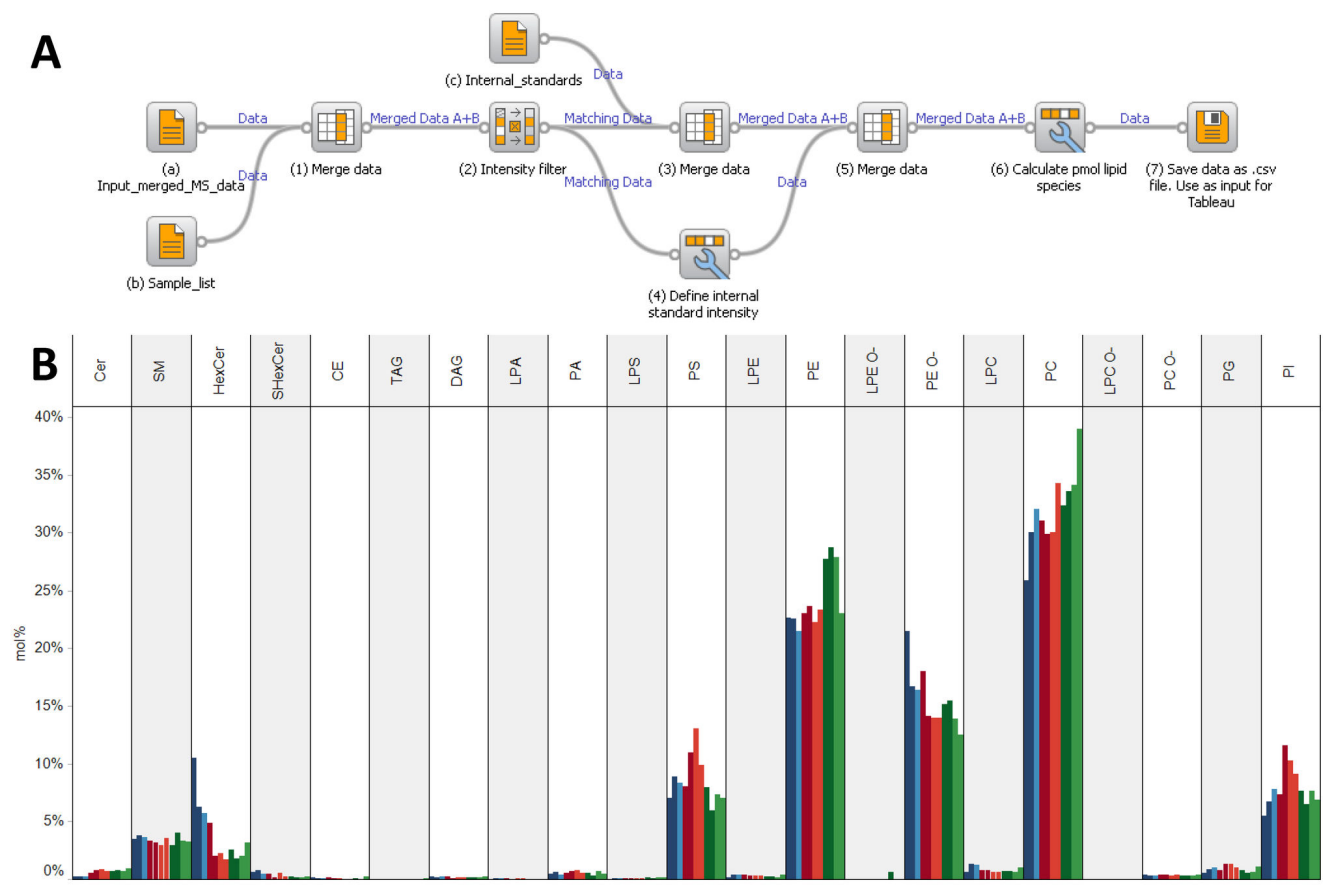
Outline of the auxiliary workflow used for lipidome data processing. (**A**) A sequence of processing steps executed by the Orange software is used to compute the molar abundance of lipid species. The processing routine utilizes three input files: (a) the output file generated by the ALEX framework specifying lipid intensities, (b) a text file specifying sample information, and (c) a text file specifying internal standards and molar spike amounts. Seven processing steps are executed in order to compute the molar abundances of lipid species for all samples. The data processing generates an output file in database table format featuring the molar abundances of lipid species, originating intensity data, all accessory lipid features and all sample information. (**B**) Lipid class composition of cerebellum, hippocampus and S1BF from wild-type and PRG-1 knockout mice as automatically calculated and displayed using Tableau Software.

In order to compute molar abundance (e.g. fmol) of lipid species we made use of the database exploration tool and open-source software Orange [28]. The molar abundance of lipid species is easily computed via a sequence of processing steps (depicted in Fig. 5). Step (1); the lipid species intensity data generated by the ALEX framework is specified as input (icon (a)) and merged with a second input specifying sample information (e.g. tissue type, genetic information, name of mice; icon (b)). Step (2); an intensity filter is implemented in order to remove intensities below a user-specified threshold if deemed necessary. Step (3); a third input specifying the spiked amount of internal standards is incorporated (icon (c)). Step (4); a new attribute is defined by specifying the intensities of internal standards. This attribute is used in step (6) for computing the molar abundance. Step (5); internal standards and corresponding intensities are defined for the subsequent calculation of molar lipid abundances. Step (6); an equation using the attributes lipid species intensity, internal standard intensity and spiked amount of internal standard is applied for calculating the molar lipid abundance [20]. Step (7); processed data is saved as a .csv output file (in database table format) that can be accessed for computation of mol% and lipidome visualization by Tableau Software. We here note that the Orange schema produced an output file of the neurolipidomics analysis featuring 997 targeted lipid species with accessory information across 56 sample injections producing a data matrix with a total of 1,050,504 data points (Data S1). Notably, managing and processing a dataset of this magnitude is poorly suited for Microsoft Excel and highlights the benefits of managing the data using a database exploration tools. 

**Figure 6:**
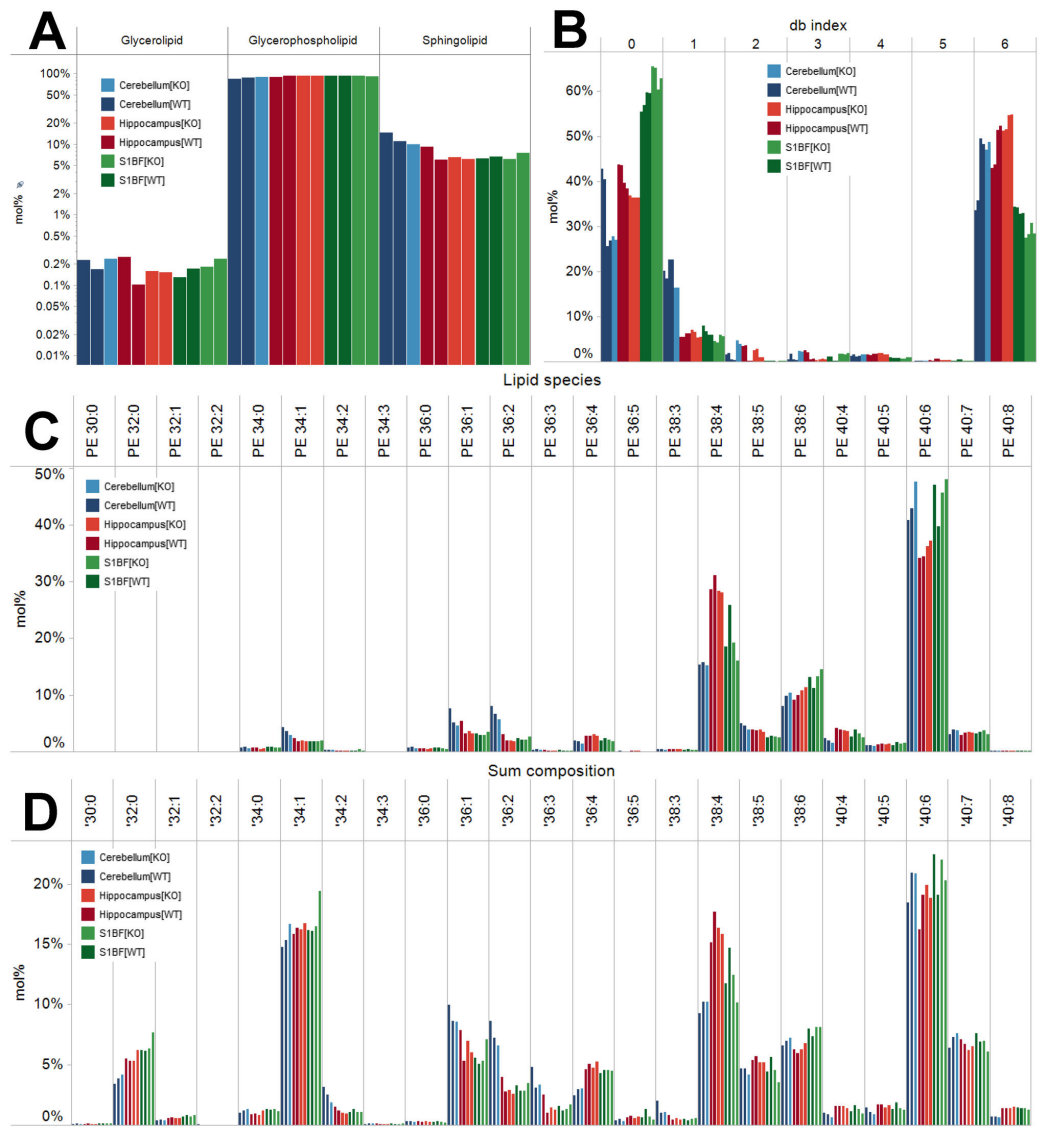
Lipidome visualization using different display formats. (**A**) mol% lipid category. Notice that the y-axis is logarithmic. Data is displayed as the average of the two technical replicates per sample. (**B**) mol% of db index of LPS species. Note that histogram include plot for both technical replicates. (**C**) mol% of PE species. Data is displayed as the average of the two technical replicates per sample. (**D**) mol% of all GPL species. Data is displayed as the average of the two technical replicates per sample. Notice that data for only one sample of cerebellum from knockout mice is available due to lack of spiked internal standards as outline in the section “Application of the ALEX software framework”. This neurolipidomics dataset is available as supporting information (Data S2).

To support rapid and efficient visualization of large lipidome datasets, we integrated Tableau Software as part of the auxiliary workflow. Tableau Software can be dynamically linked to the Orange output files such that any potential modifications within the data processing procedure can be visualized simply by updating the Orange output file and the link to Tableau. Notably, the Tableau software includes a feature that easily allows calculation and display of “mol% of lipid species” normalized to any given set of attributes and accessory lipid features in the input file. As such, the user can rapidly display the “mol% of all monitored PE species” (Figure 6C) or “mol% of all monitored glycerophospholipid species” (Figure 6D). We note, that calculation of such data formats would require implementation of additional processing steps within Orange (and equally in Microsoft Excel) in order to calculate and output such data values. We also note that these accessory lipid features provided by the ALEX framework, and consequently the information content in database table format, facilitates lipidome visualization by Tableau software.

### Application of the auxiliary workflow

To illustrate the efficacy of the auxiliary workflow we here present the results of the neurolipidomic pilot study. First, we assessed the lipid class composition of the three mouse brain tissues from wild-type and PRG-1 knockout mice (Figure 5B). The lipid class composition of brain tissues was primarily comprised of PC, PE, PE O-, PS, PI, SM and HexCer lipids. In addition, the analysis also showed a low abundance of Cer, SHexCer, CE and TAG species (Figure 5B). This result corroborates previous reports on brain lipid composition [36–38]. Notably, comparing the lipid class composition of the brain tissues did not show any pronounced differences between the wild-type and PRG-1 knockout mice. Moreover, the analysis also did not show any major difference in lipid class composition between the three brain tissues. To further interrogate the lipidome data, we explored alternative display formats including lipid category composition (e.g. glycerophospholipids, sphingolipids and glycerolipids, Figure 6A), lipid species composition within a defined lipid class (e.g. PE species, Figure 6C), lipid species composition across a defined lipid category (e.g. all glycerophospholipids, Figure 6D) and db index within a defined lipid class (e.g. LPS, Figure 6B). Using the various modes of lipidome visualization, we observed that each of the three mouse brain tissues featured specific signatures of lipid species. Specifically, we observed that the composition of PE species was different between all three tissues; the hippocampus comprised a relatively high level of PE 38:4, the S1BF contained relatively high levels of PE 40:6, and the cerebellum contained a relatively high level of PE 40:6 and minor but systematically higher levels of PE 34:1, PE 36:1 and PE 36:2 as compared to the two other tissues. These distinct lipidome hallmarks could also be observed when assessing the collective lipid species composition across all glycerophospholipids (Figure 6D) albeit with a less pronounced differences as compared to only PE species. The specific lipid compositions of the three brain tissues were further interrogated by visualizing the distribution of double bonds within the fatty acid moieties of LPS species. This visualization revealed that the S1BF comprised relatively high levels of LPS species with 0 double bonds (primarily attributed LPS 18:0) being offset by lower levels of LPS species having 6 double bonds (attributed LPS 22:6). In comparison, the cerebellum showed a characteristic distribution of LPS species with higher levels of species with 1 double bond as compared to the two other tissues. The LPS composition of the hippocampus comprised a double bond distribution intermediate of the S1BF and the cerebellum. We note that the lipidome visualization did not reveal any pronounced differences in lipid species composition between any of the tissues from wild-type and PRG-1 knockout mice. This observation is furthermore substantiated by results of principal component analysis (data not shown). 

## Conclusions

Here we presented a platform for streamlined processing, computation and visualization of high-content lipidomics datasets acquired using high-resolution Orbitrap mass spectrometry. The platform features a novel routine for querying proprietary spectral data that supports identification and quantification of lipid species, execution of quality control routines and rapid visualization of lipidome data. The platform utilizes three software modules: the ALEX framework that accesses and queries mass spectral data, the visual programming tool Orange that integrates sample information and computes molar lipid abundances, and the visual analytical tool Tableau Software for lipidome visualization. A key asset of the framework is the storage of lipidome data in “database table format” that enables using a multitude of data attributes for robust data processing and visualization. To demonstrate the efficacy of the platform, we presented a comparative neurolipidomic pilot study of mouse cerebellum, hippocampus and S1BF from wild-type and PRG-1 knockout mice [29]. The analysis demonstrated a distinct lipid species signature for each of the three brain tissues, but failed to ascertain any pronounced perturbations of lipid composition in mice devoid of the PRG-1 gene. This observation can potentially be explained by the localized expression of PRG-1 in the postsynaptic density of glutamatergic synapses. Regional differences in the lipidome composition induced by PRG-1 deficiency might potentially be concealed by the complexity of the macroscopic brain tissues investigated herein. We note that the ALEX framework can easily be adapted for processing of high-resolution shotgun lipidomics data acquired by any type of instrumentation (e.g. LTQ FT, Q Exactive, Orbitrap Fusion, solariX) provided spectral peak lists are stored in .txt file format. Moreover, the Orange processing procedure and Tableau visualization can be extended to include various filters for improved lipid identification, to calculate molar abundance of lipid species per unit of sample material (e.g. pmol lipid/µg protein), to perform statistical testing or multivariate analysis, and to integrate and support processing and visualization of lipidome data acquired by MS/MS analysis. Notably, the auxiliary workflow can also be adapted to use other database-orientated exploration tools than Orange and Tableau software. Finally, we argue that storage of lipidomics data in database table format can be a future avenue for data dissemination since it enables investigators to easily access, inspect and apply such resource data. 

## Supporting Information

Data S1 **Processed neurolipidomics data including fmol lipid species.** The data is organized in database table format.
(CSV)

Data S2 **Processed neurolipidomics data in Tableau file format.** The file can be opened and navigated using the freeware Tableau Reader (www.tableausoftware.com/products/reader).
(ZIP)
