# Correction: Analysis of Lipid Experiments (ALEX): A Software Framework for Analysis of High-Resolution Shotgun Lipidomics Data

**DOI:** 10.1371/journal.pone.0093351

**Published:** 2014-04-02

**Authors:** The PLOS ONE Staff

## Notice of Republication

This article was republished on April 2, 2014, to replace an incorrectly published version. Please download this article again to view the correct version.
